# Functional Interface Modifier with Visualizations of Dendrite Growth and Heat Evolution in Lithium Metal Batteries

**DOI:** 10.1021/acsami.5c25165

**Published:** 2026-06-23

**Authors:** Bereket Woldegbreal Taklu, Tsung-I Yeh, Ashok Vallal Saravanan, Elango Balaji Tamilarasan, Siyanand Kumar Chaudhary, Zabish Bilew Muche, Boligarla Vinay, Teshome Gonfa Hordofa, Kidane Goitom Gerezgiher, Chung Ray Weng, Tzu-Ting Hung, Sheng-Chiang Yang, Wei-Nien Su, Bing Joe Hwang

**Affiliations:** † Nano-electrochemistry Laboratory, Department of Chemical Engineering, 34878 National Taiwan University of Science and Technology, Taipei 106, Taiwan; ‡ Sustainable Electrochemical Energy Development Center, 34878 National Taiwan University of Science and Technology, Taipei City 106, Taiwan; § Battery Research Center of Green Energy, Ming Chi University of Technology, New Taipei City 24301, Taiwan; ∥ Nano-electrochemistry Laboratory, Graduate Institute of Applied Science and Technology, 34878 National Taiwan University of Science and Technology, Taipei 106, Taiwan; ⊥ National Synchrotron Radiation Research Center (NSRRC), Hsin-Chu 30076, Taiwan

**Keywords:** dendrite visualizations, heat evolution, artificial interface stabilizations, solvent-free approach, Li−Sn alloying

## Abstract

For high-energy-density batteries, the lithium–metal anode remains the ultimate choice. However, lithium dendrites pose a serious safety concern for the practical use of lithium anodes. Here, a multipurpose artificial passivation is fashioned on the lithium–metal interface via a facile, versatile approach using stannic chloride as a sacrificial agent. Bilayered structure, rich in LiCl, coupled with a Li–Sn alloy, reinforces uniform lithium flux and suppresses electrolyte decomposition. The structure promotes uniform deposition and dendrite-free lithium growth. The protected anode operated at a high current density of 5 mA cm^–2^/5 mAh cm^–2^ and demonstrated cycling for more than 600 h. Operando confocal OM-based visualization of lithium growth phenomena and heat evolution measurements at the molecular level reveal dendrite-free lithium deposition and electrolyte decomposition, respectively. An in situ EIS measurement shows mitigation of the interfacial reaction. Moreover, the electrochemical performance of the battery achieves capacity retention of 97.1% (after 360 cycles at 0.5 mA cm^–2^), 100.3% (after 410 cycles at 3 mA cm^–2^), and 93.8% (after 480 cycles at 5 mA cm^–2^), respectively, with a LiFePO_4_ cathode for Sn–Li metal anode. This approach demonstrates a solvent- and binder-free method for stabilizing the lithium surface, enabling guided lithium growth for safe, high-performance lithium–metal anodes.

## Graphical abstract

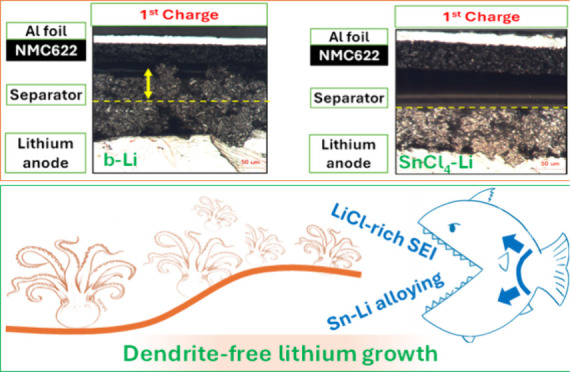

## Introduction

To address the energy demand and mitigate the effects of excessive fossil fuel consumption, batteries with lower costs and higher energy densities are essential.^1–5^ Despite their reliance on intercalation electrodes, conventional lithium-ion batteries have low energy density.^6–9^ By coupling with a holy grail lithium with the lowest electrode potential (−3.04 V vs SHE) and the highest capacity (3860 mAh g^–1^) advances the energy density.^7,10–13^ Nonetheless, the primary downside of lithium metal is its inherent characteristics, including lithium thermodynamic reactivity, solid–electrolyte interface (SEI) crack, and random Li nucleation, which result in low initial Coulombic efficiency (iCE), in addition to safety concerns from dendrite growth.^4,14–18^ As interface stabilizers, solvent-dependent metallic chloride salts, M_*x*_Cl_*y*_ (M = Cu, Zn, In, Al, Bi, As, Ga, and Si), contribute to alleviating interface challenges and monitoring the lithium flux.^19–23^ However, the approach utilized a variety of solvents, including THF, DOL, NMP, and DME, as a medium in spin-coating^19^ or drop-casting^20^ for lithium metal surface modification. A mixed interlayer formation comprising electronically conductive Li–M alloys and ionically conductive LiCl at the lithium interface overcomes nonuniform deposition and dendrite growth. The issue ofsolubility of metallic salts, solvent toxicity, and compatibility with lithium metal all depend heavily on the choice of the suitable solvents. Lithium stabilization as a solvent-free or binder-free approach advances cost-effectiveness, scales up feasibility, and avoids the use of hazardous chemicals.^24^ These approaches can be used in both the liquid and gaseous states. Gas-phase treatment of lithium via I_2_(g), S_8_(g), N_2_(g), Freon R-134a (1,1,1,2-tetrafluoromethane), and NH_4_F results in LiI, Li_2_S, Li_3_N, LiF, as well as LiF, Li_3_N, Li_2_NH, LiNH_2_, and LiH formation as SEI components, respectively.^25–30^ An electrolyte additive, such as C_2_Cl_4_, induced in situ LiCl-rich SEI in the organic liquid electrolyte,^31^ and solid electrolyte (halide SE, Li_3_InCl_6_) as an electrolyte additive was also used to enhance antipulverization properties to mitigate dead lithium metal and dendrite formation.^32,33^

In this work, we present a multipronged strategy for lithium stabilization using stannic chloride (SnCl_4_) as a liquid salt to enable chlorination and alloy formation, thereby enhancing the longevity of lithium metal batteries. Operando visualization of the dendrite shows Li deposition with a dense microstructure and fast lithium diffusion induced by Sn–Li alloying, as evidenced by operando confocal OM. The interface parasitic reaction and electrolyte decomposition, evaluated at the molecular level by operando heat evolution using a TAM-IV microcalorimeter, validating the suppression of parasitic reactions and enhanced stability at the electrolyte/electrode interface. Moreover, an electrochemical cell confirms the substantial improvement on the interface alteration evidenced by outstanding capacity retention of 97.1% (360 cycles at 0.5 mA cm^–2^), 100.3% (410 cycles at 3 mA cm^–2^), and 93.8% (480 cycles at 5 mA cm^–2^), respectively, when coupled with a LiFePO_4_ cathode. Under LiNi_0.6_Mn_0.2_Co_0.2_O_2_ cathodes in a carbonate electrolyte, enhanced cycling stability is achieved.

## Experimental Sections

Stannic chloride (SnCl_4_, 98%) is used as a sacrificial interface stabilizer, and approximately 2–3 μL is applied to lithium after rolling. The prepared lithium metal was then cut into 16 mm discs, and all subsequent processes were carried out in an argon-filled glovebox (MBraun, 2020). Symmetric- and asymmetric-cell tests were conducted at different current densities to demonstrate the improvement. For a symmetric cell, current density from 0.1 to 5 mA cm^–2^ was used at a fixed 1 mAh cm^–2^ capacity. Additionally, the high-current durability test was operated at 5 mA cm^–2^/5 mAh cm^–2^ for both bare and treated lithium metal anodes using a commercial ether-based electrolyte, 1 M LiTFSI in DOL/DME + 2 wt % LiNO_3_ (1:1 v/v). Additionally, carbonate electrolytes, including 1 M LiPF_6_ EC–DEC, were used to demonstrate the versatility of the artificial interface SEI at 1 mA cm^–2^/1 mAh cm^–2^. Cyclic voltammetric (CV) measurements were performed for symmetric cells (x-Li/x-Li) and LiFePO_4_/x-Li (x = b or Sn) using both electrolyte systems. Additionally, full-cell tests were conducted at different current densities for both LiFePO_4_ and LiNi_0.6_Mn_0.2_Co_0.2_O_2_ cathodes, with bare lithium (b-Li) and SnCl_4_-treated lithium (Sn–Li) as the anode. Current densities of 0.1–5 mA cm^–2^ were applied to both anodes to assess the benefits of artificial SEI formation and to promote interface stabilization. An electrolyte volume of 60 μL was taken from 1 M LiTFSI in DOL/DME + 2 wt % LiNO_3_ (1:1 v/v), and 1 M LiPF_6_ EC–DEC electrolytes were used to assemble with LiFePO_4_ and LiNi_0.6_Mn_0.2_Co_0.2_O_2_ cathodes, respectively, in GB with O_2_ and H_2_O amounts <0.1 ppm.

Operando visualization of lithium growth was investigated using a confocal optical microscope (confocal OM) in a full-cell configuration (Li|LiNi_0.6_Mn_0.2_Co_0.2_O_2_) with 600 μL of 1 M LiPF_6_ EC–DEC electrolyte, integrated with AutoLab. A current density of 0.5 mA cm^–2^ was used to observe lithium dendrite growth and deposition phenomena. Likewise, operando heat-evolution measurements were performed by integrating AutoLab with a battery-cycler microcalorimeter (TAM-IV) at 25 °C, using five cycles in an x-Li|Cu (x = b or Sn) cell with 60 μL of 1 M LiPF_6_ EC–DEC electrolyte. Using the LiNi_0.6_Mn_0.2_Co_0.2_O_2_/x-Li (x = b or Sn) system, the cell was scanned at 0.2 mV s^–1^ from 2.7 to 4.3 V vs Li/Li^+^ under CV, and heat flow was recorded at every point during cycling. Additionally, the detailed methodology for DFT studies is stated in the Supporting Information.

## Results and Discussion

For ex situ artificial stabilization, a rolled and polished lithium metal foil was uniformly chlorinated using stannic chloride (SnCl_4_) as the sacrificial liquid salt via a solid–liquid-dependent reaction on the Li metal surface. A brush-coating approach under a solvent- and binder-free protocol is introduced. Approximately 2 μL of liquid salt is used in lithium treatment to form an artificial protective layer, denoted as Sn–Li metal. The glossy, silvery lithium surface instantly changed due to a rapid displacement reaction. As examined by XRD in Figure 1a, the surface reaction product on Sn–Li surfaces revealed LiCl and metallic tin (Sn), which were identified as major products within the scanned 2θ range. The principal characteristic peaks of metallic tin, detected at around 31° 2θ, were also observed; hydrated lithium chloride (LiCl·H_2_O) was identified at 22.4°. Lithium chloride, LiCl, diffraction peaks were pinpointed at 34.8°, 38.4°, and 50° 2θ angle, respectively.^34,35^ The peak intensity is mainly related to the thickness of the surface passivation layers formed by the reaction products on the lithium surface. Morphological examination of the treated surface reveals a transformation of the lithium foil, which is entirely covered by the reaction products, as indicated by elemental mapping and surface morphological changes (Figure 1b). Additionally, SEM cross-sectional examination revealed the compositional distribution of LiCl and Sn across the treated lithium metal surface horizon. Figure S1 shows the energy-dispersive X-ray spectroscopy (EDS) analysis of the treated lithium metal surface, with identified peaks for Sn and Cl. The schematic illustration in Figure 1c demonstrates the surface treatment of lithium foils via brush coating and its constructive impact during cell operation. The LiCl-rich SEI and Sn metal synergistically suppress electrolyte decomposition and monitor lithium flux, enabling dendrite-free lithium deposition and thereby contributing to superior cell performance.

**Figure 1:**
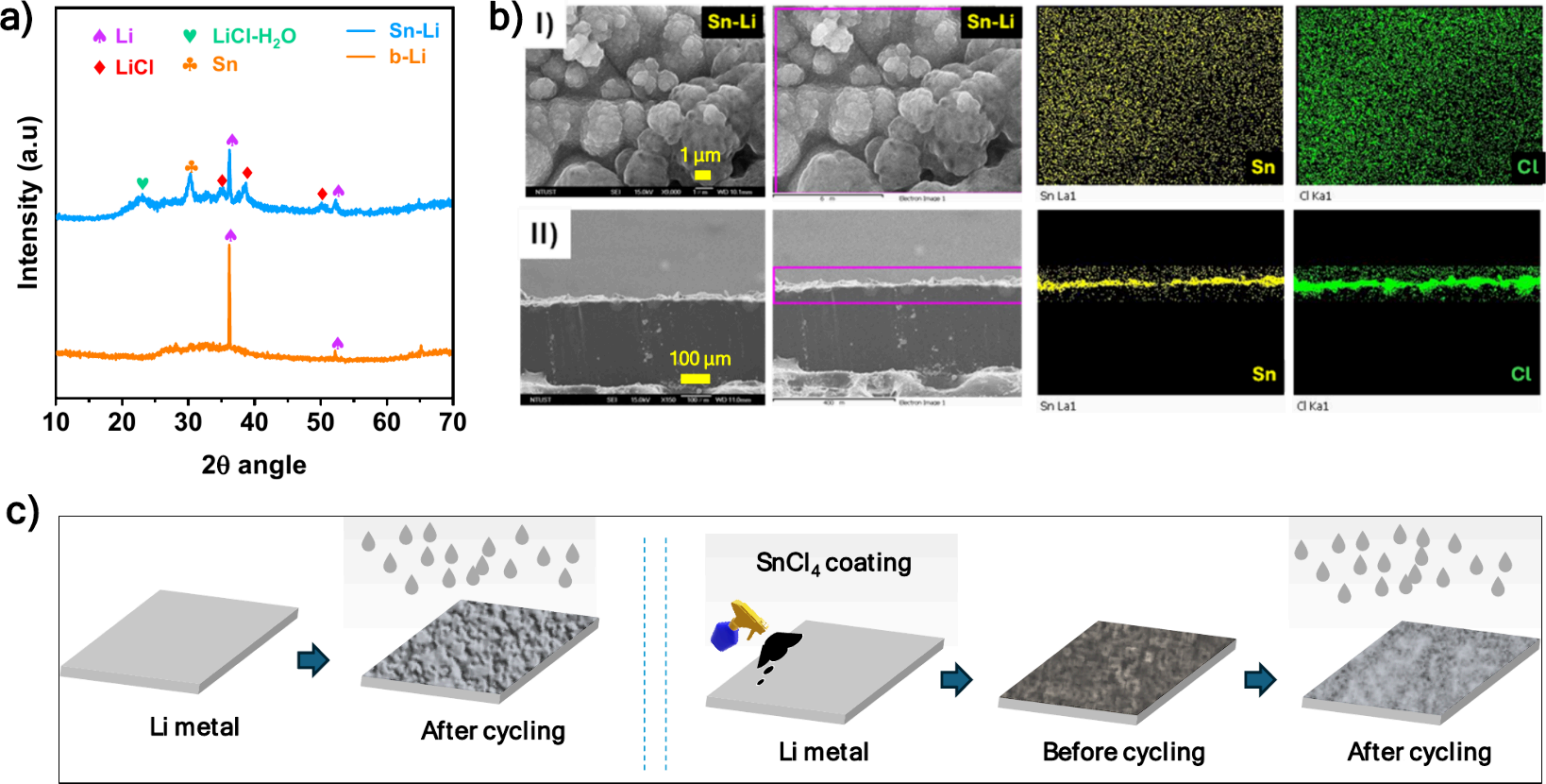
Lithium foil surface treatment and material characterization for b-Li and Sn–Li metal anode. (a) XRD pattern of lithium metal before and after surface treatment. (b) SEM and cross-sectional view of lithium metal before and after treatment, including elemental analysis via EDS mapping. (c) Schematic illustration of bare lithium metal and lithium treatment via liquid chloride salt, SnCl_4_, using brush coating and its benefits during cell operation for long-term cyclic performances.

Additionally, the surface chemical composition of the treated lithium metal samples was exclusively examined by XPS. The treated lithium, Sn–Li, indicates the doublet peaks in Cl­(2p) identified as (201.1 and 202.6 eV).^30,36,37^ The reaction product from Sn, as in situ seeding, indicated at Sn­(3d) peaks, spotted (484.9 and 493.9 eV),^24,34,35^ as shown in Figure S2. To explore the synergistic contribution of the LiCl-rich artificial protective layer and highly lithiophilic tin metal, a galvanostatic symmetric cell with a fixed 1 mAh cm^–2^ areal capacity was tested at different current densities (0.1–5.0 mA cm^–2^). The protected lithium, Sn–Li, reveals outstanding cyclability over a range of step current densities and operated for more than 700 h. at 2 mA cm^–2^/1 mAh cm^–2^, as shown in Figure 2a and its inset. Untreated lithium metal, b-Li, exhibits severe voltage hysteresis, with a gradual voltage drop as the current density stepwise increased to 2 mA cm^–2^ due to dendritic lithium deposition, leading to battery failure before reaching 400 h under a conventional ether electrolyte, 1 M LiTFSI in DOL/DME + 2 wt % LiNO_3_ (1:1 v/v), as indicated in Figure 2a. Moreover, Figure S3 shows the Sn–Li/Sn-Li cell operated at 0.5 mA cm^–2^/1 mAh cm^–2^ operated for 500 h under 1 M LiTFSI in DOL–DME + 2 wt % LiNO_3_ electrolytes. A long-term high-current-density and areal-capacity cell test was conducted for b-Li and Sn–Li at 5 mA cm^–2^/5 mAh cm^–2^. The protected lithium (Sn–Li) operated smoothly for more than 600 h. Notably, for b-Li, it can only operate for 200 h, as followed by a short circuit, as indicated in Figure 2b.

**Figure 2:**
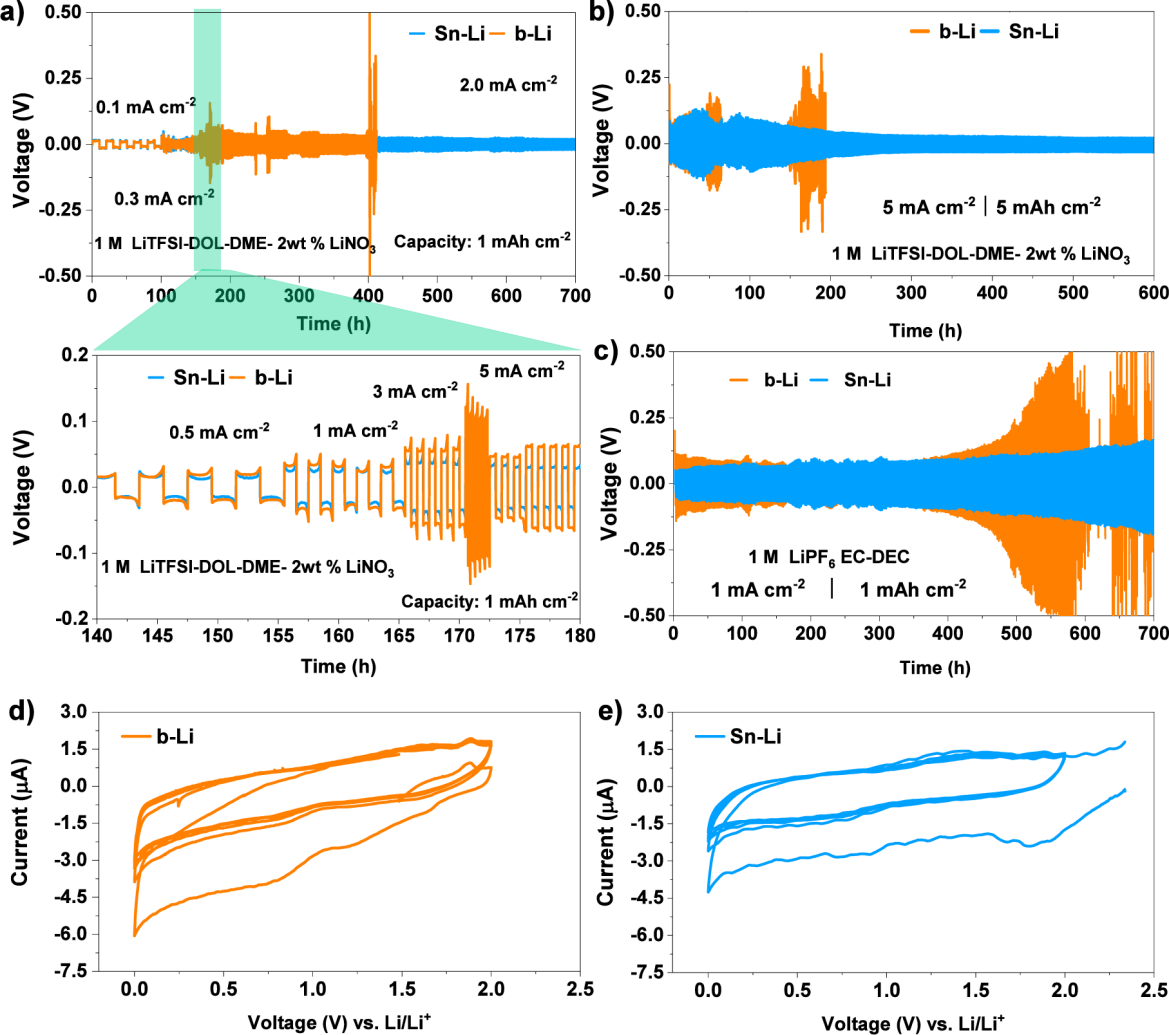
Galvanostatic symmetric-cell cyclability under b-Li, and Sn–Li, in conventional ether electrolyte, 1 M LiTFSI DOL/DME + 2 wt % LiNO_3_ (1:1 v/v) and carbonate electrolytes 1 M LiPF_6_ EC–DEC. (a, b) Symmetric cells operated in an ether electrolyte at different current densities and areal capacities; (c) symmetric cell at 1 mA cm^–2^ with 1 mAh cm^–2^ areal capacity for carbonate-based electrolyte for both Li-electrodes. CV measurement for asymmetric cells, Li/Cu, showing electrolyte decomposition for (d) bare lithium, b-Li, and (e) treated lithium, Sn–Li, respectively.

To explore more on the versatility of the coating, a carbonate-based electrolyte, 1 M LiPF_6_ EC–DEC, was employed, as shown in Figure 2c. The cell was tested at a high current density and capacity of 1 mA cm^–2^/1 mAh cm^–2^, highlighting interfacial stabilization of coating material and its dendrite-suppression capability. The corrosive behavior of the carbonate electrolyte leads to rapid polarization in the Li||Li cell, causing b-Li to experience a short circuit before reaching 600 h. In contrast, the treated lithium anodes demonstrated superior stability, without signs of short-circuit formation, for up to 700 h at lower overpotentials. Furthermore, the stabilization of the Li/electrolyte interface and electrolyte decomposition were studied using CV measurements in a symmetric-cell configuration at a scan rate of 0.1 mV s^–1^. It shows that the protected Sn–Li anode fully mitigates the interfacial reaction compared to the bare b-Li anode, as shown in Figure 2d,e. Significant stabilization of the Li/electrolyte interface and a decrease in the current signal indicate reduced overpotential growth in Sn–Li relative to that in b-Li, consistent with symmetrical cell tests at different current densities.

Further demonstrates the alloying–dealloying process (Figure S4) in an asymmetric cell, Li/Cu, was assembled using an electrolyte of 1 M LiPF_6_ in EC/DEC, with the addition of 3% v/v SnCl_4_ liquid salt. It is interesting to note that an in situ Sn–Li alloy formation peak appears as the cell approaches 0 V, accompanied by an additional peak from SEI formation. The peak current for SEI formation is observed at a higher voltage (>2 V), indicating passivation of the lithium surface by the liquid salt. It is important to observe SEI formation in an SEI using LiCl. LiF- and LiCl-rich SEI, together with Sn–Li alloying ability, are attributed to the enhanced cycle life even under high-current-density cell operation. Interestingly, the Sn alloying and dealloying peaks were observed for the cell, as it was scanned from 0 to 2 V vs Li/Li^+^.

Besides, DFT calculations, as described in the Supporting Information, were used to illustrate alloying. To evaluate lithium-ion mobility in the selected materials, the climbing-image nudged elastic band (C-NEB) method was used to calculate the activation energy barriers (*E*_a_) based on their most stable surface terminations. After refining the surface models, C-NEB simulations indicated a notably reduced diffusion barrier for Li_5_Sn_2_ at 0.241 eV compared to 0.433 eV for pure Li. This decreased barrier in Li_5_Sn_2_ results from its strong lithophilicity, which facilitates lithium-ion mobility, thereby increasing Li^+^ conductivity and enhancing lithium diffusion, as illustrated in Figure S5.

### Operando Confocal OM for Visualization of Lithium Deposition and Growth Phenomena

To comprehend the practicability of protected lithium, b-Li and Sn–Li, the lithium deposition and growth mechanism was elucidated using a full cell, LiNi_0.6_Mn_0.2_Co_0.2_O_2_/x-Li (x = b or SnCl_4_), with a commercial carbonate electrolyte, 1 M LiPF_6_ EC-DEC, at a current density of 0.5 mA cm^–2^. Lithium deposition on the bare lithium anode, b-Li, shows significant volume expansion and considerable mossy dendritic lithium. Porous and fluffy lithium trundled over the lithium surface results in dendritic lithium growth and soft short circuits, as illustrated in Figure 3a,b. The lithium growth phenomenon at a state of charge was explored by using an operando confocal image. At open-circuit voltage (OCV), the separator was positioned at the center for both cell configurations. At the early stage of lithium growth, noticeable dendritic growth was observed for the b-Li anode. However, lithium growth in Sn–Li is more evenly distributed across the lithium surface. Interestingly, at 50% state of charge (SOC) of the NMC622 cathode, very clear, aggressive dendritic lithium growth was noticed for b-Li. In a fully charged state, the separator is pushed toward the cathode side, facilitating short-circuit formation and cell failure; in the discharge state, mossy and dendritic lithium have poor reversibility, as shown in Figure 3a. The separator’s original position was displaced toward the cathode because of the significant volume expansion and nonuniform lithium deposition, resulting in a short circuit. The displacement in the position of the separator is indicated by an arrow in the image at different states of charge, as seen in Figure 3a,b.

**Figure 3:**
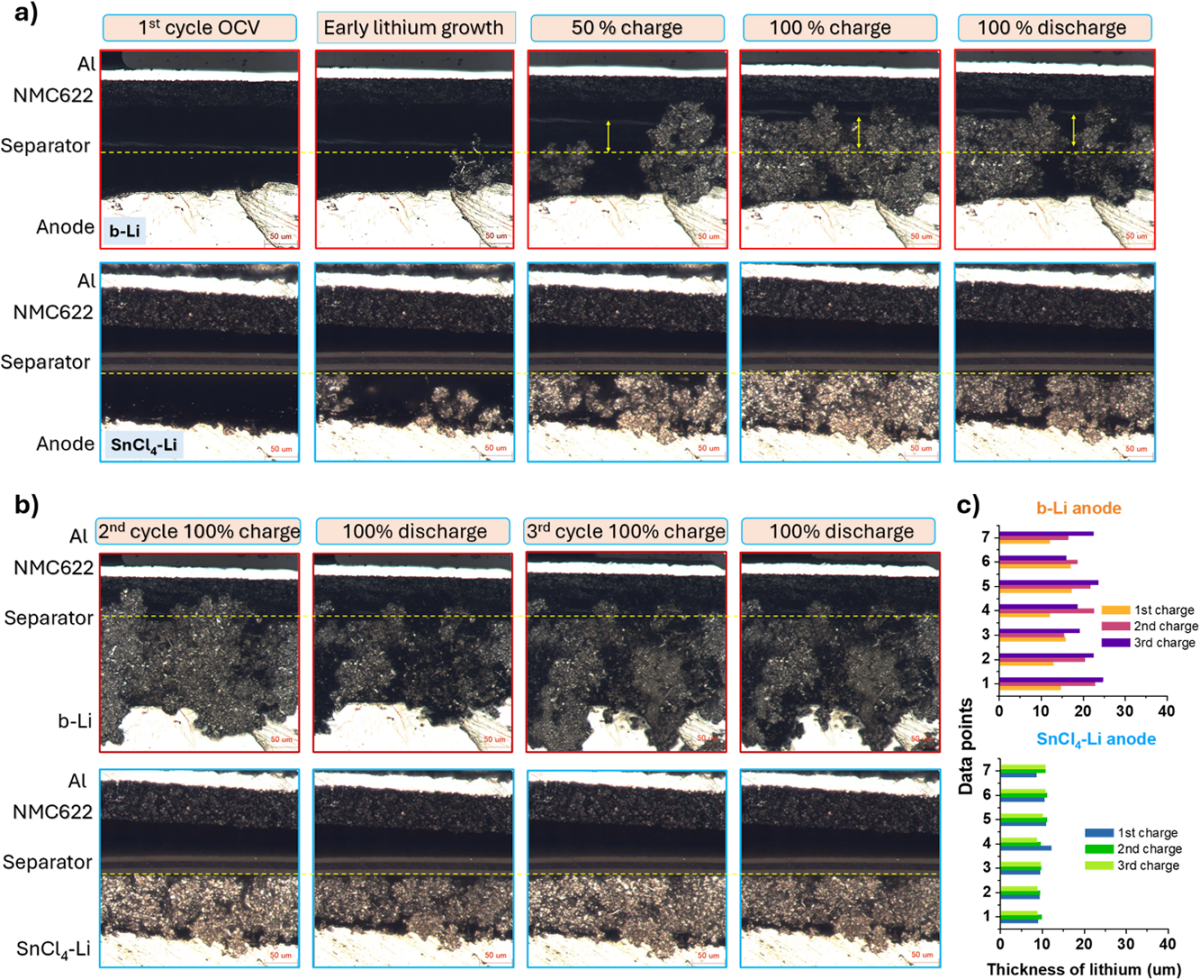
Operando confocal microscopic observation for the lithium growth and thickness of deposited lithium under a full-cell configuration, LiNi_0.6_Mn_0.2_Co_0.2_O_2_/x-Li (x = b or Sn) at 0.5 mA cm^–2^ using a carbonate electrolyte, 1 M LiPF_6_ in EC-DEC. Panels (a) and (b) show the SOC of the cathode for bare lithium anode for at OCV, first cycle, second cycles, and third cycles, and (c) thickness of deposited lithium for fully charged cathode for first, second, and third cycles.

Noteworthy improvements in dense lithium deposition, overcome volume expansion during charging, and enhanced lithium reversibility were observed after the SnCl_4_ treatment of lithium metal. The separator maintained its position even after being fully charged, indicating that its dense microstructure and dendrite-free lithium deposition enable it to remain in its original position at the OCV state, as shown by the dashed line. Operando confocal OM visualization of lithium growth in a full cell was also supported by video evidence. The protective layer in Sn–Li promotes lithium growth with more compact deposition and enhanced cyclability, as evidenced in full cell tests. Volume expansion is a major issue in lithium-ion batteries, particularly with the Si anode.^38^ Lithium also undergoes volume expansion, leading to continuous SEI cracking and electrolyte decomposition, which significantly affects battery cycle life. The contributing factors, including a LiCl-rich SEI with in situ nucleation seeds, Sn on the lithium surface, are attributed to dendrite-free lithium growth. The compactness of lithium deposition in Sn–Li is much better than that of b-Li. As a result, such dense lithium growth and dendrite-free deposition significantly contributes to the batteries’ superior performance.

**Figure 4:**
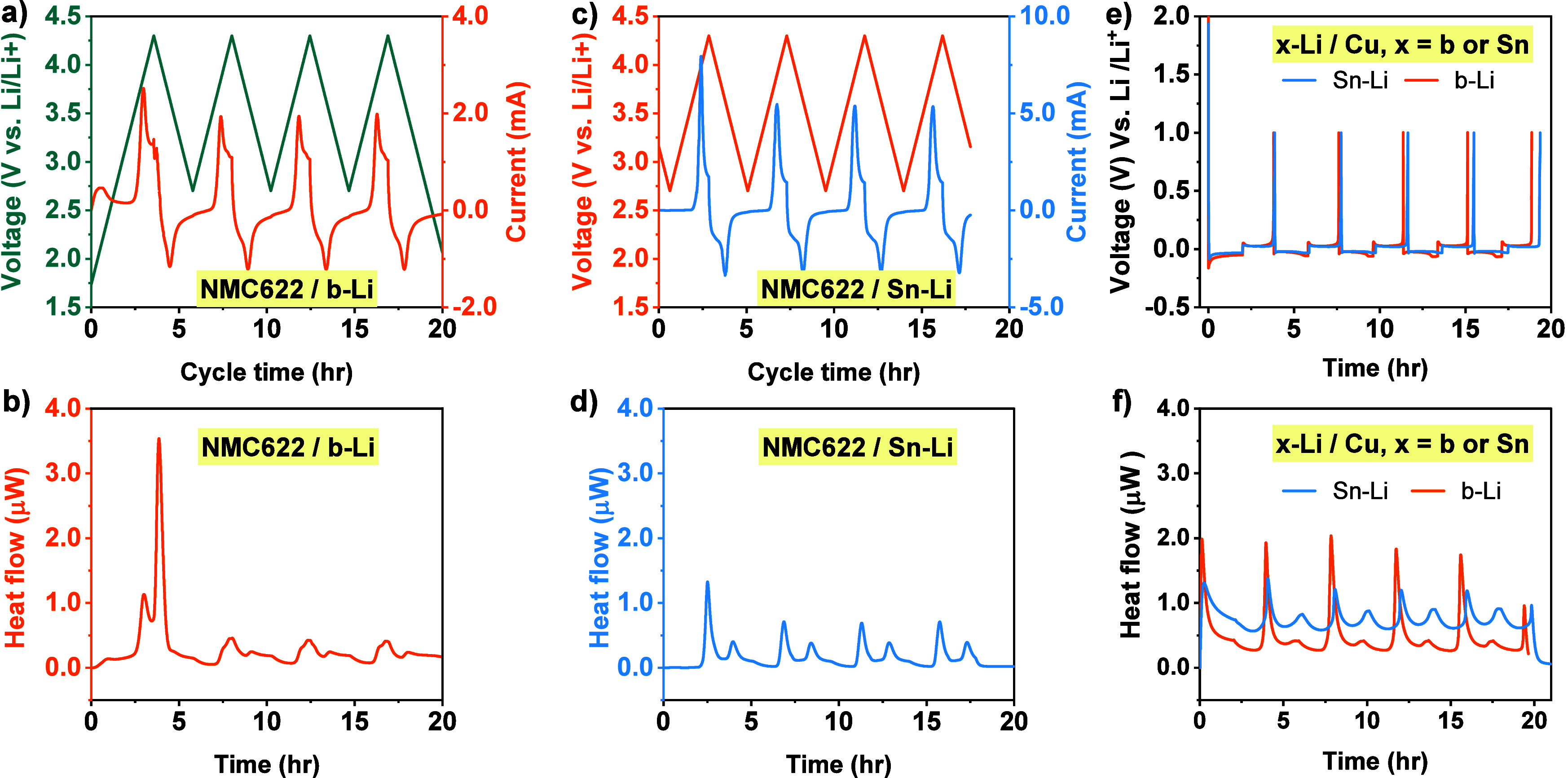
Operando heat-evolution measurement for the extent of parasitic reaction at the LiNi_0.6_Mn_0.2_Co_0.2_O_2_-electrolyte and x-Li-electrolyte (x = b or Sn–Li) interface. Panels (a) and (b) represent the voltage–current plot and heat evolution for b-Li anode, respectively, and panels (c) and (d) represent the voltage–current plot and heat evolution for Sn–Li anode, respectively. Panels (e) and (f) show the asymmetric cell (Li/Cu) under carbonate electrolyte, 1 M LiPF_6_ in EC–DEC.

### Operando Heat Evolution and Parasitic Interface Reaction at the Electrode Surface

Parasitic reactions and electrolyte decomposition on the lithium metal anode were studied as causes of electrolyte consumption and continuous resistance growth at the electrode via operando heat-evolution measurements, as cumulative heat release.^24^ CV measurements at a scan rate of 0.2 mV s^–1^ were used to evaluate heat evolution associated with interfacial reactions, as shown in Figure 4a-d. The heat evolution shows two principal peaks, at approximately 3.9 V for the oxidation current and 3.6 V for the reduction current, indicating electrolyte decomposition at the cathode and anode, respectively. For LiNi_0.6_Mn_0.2_Co_0.2_O_2_/b-Li at 3.9 V vs Li/Li^+^, a high oxidative current on the cathode is observed, accompanied by a heat-evolution peak, indicating electrolyte decomposition on the cathode surface. Across the voltage array at 3.6 V vs Li/Li^+^, a higher reduction current was observed at the heat-evolution peak, indicating electrolyte decomposition at the lithium surface. Interestingly, the heat evolution observed during reduction is higher than that during oxidative heat release in the LiNi_0.6_Mn_0.2_Co_0.2_O_2_/b-Li cell as shown in Figure 4a,b. It indicates a significant interfacial reaction at the Li/electrolyte interface during the first cycle, driven by lithium corrosion from the aggressive carbonate electrolyte. The consecutive heat cycles associated with the Li/electrolyte interface decrease; however, heat still evolves because of an interfacial reaction at the b-Li anode. On the other hand, LiNi_0.6_Mn_0.2_Co_0.2_O_2_/Sn–Li exhibits a similarly intense heat-evolution peak at approximately 3.9 V during cathodic electrolyte decomposition, accompanied by high heat release. The heat evolution from the cathode is higher than that from the anode, observed around 3.6 V, indicating that the primary electrolyte consumption occurs at the cathode, as indicated in Figure 4c,d. Artificial passivation in Sn–Li effectively mitigates electrolyte decomposition at the lithium surface, and a LiCl-rich SEI further mitigates it. However, the major heat release originated from the cathode side, possibly due to continuous capacity fading and a short cycle life in a 1 M LiPF_6_ EC-DEC carbonate-based electrolyte for lithium metal batteries. To avoid interference from the cathode, an asymmetric cell (x-Li/Cu, x = b or Sn) operated at 0.5 mA cm^–2^ to exhibits interfacial reactions at the Li/electrolyte interface for both bulk and deposited lithium metal. Interestingly, the lithium reversibility and heat evolution from interfacial reactions show significant improvement after lithium protection, as depicted in Figure 4e,f. The reduction of heat flow from the interfacial reaction is attributed to the interface’s robustness against electrolyte decomposition.

### Full-Cell Performance in Lithium Metal Batteries

Despite its high energy density, inadequate protection of the lithium anode results in electrolyte consumption and dendritic lithium growth. An artificial passivation rich in LiCl and Sn effectively suppresses dendritic lithium growth, serving as a nucleation site, and boosts cyclability. Figure 5 shows the long-term cycling performance of Sn–Li at a high current density, performed with LiFePO_4_ as the cathode at 3 and 5 mA cm^–2^. The initial Coulombic efficiency (iCE) of the full-cell battery, coupled with a LiFePO_4_ cathode, at 3 mA cm^–2^ was 88.5% for the b-Li anode and 91.7% for the Sn–Li anode. The protective layer primarily improves interfacial stability and suppresses parasitic reactions that cause continuous SEI cracking and lithium dendrite formation. Cycling performance of b-Li with a LiFePO_4_ cathode, shown in Figure 5a,b, was operated for 410 cycles; however, the cell’s CE and capacity declined after 280 cycles. Interestingly, the cell was operated for over 410 cycles with Sn–Li anode, achieving a retention capacity exceeding 100.3% at 3 mA cm^–2^, as shown in Figure 5c,d. The average Coulombic efficiency (ACE) after 410 cycles is 99.76% for Sn–Li, indicating better cyclability than 88.9% in the b-Li anode at the same cycle number. For a high current density cell, 5 mA cm^–2^, the Sn–Li delivers a superior retention capacity of 93.8% after 480 cycles. The bare lithium foil, however, short-circuited at 5 mA cm^–2^ after 200 cycles, indicating that the Sn–Li alloying and a LiCl-rich SEI facilitate lithium deposition and stripping phenomena. The surface of a bare lithium foil is affected by electrolyte decomposition, and continuous SEI cracks lead to dendritic lithium deposition at high current densities, causing short-circuiting under symmetric and full-cell conditions.

**Figure 5:**
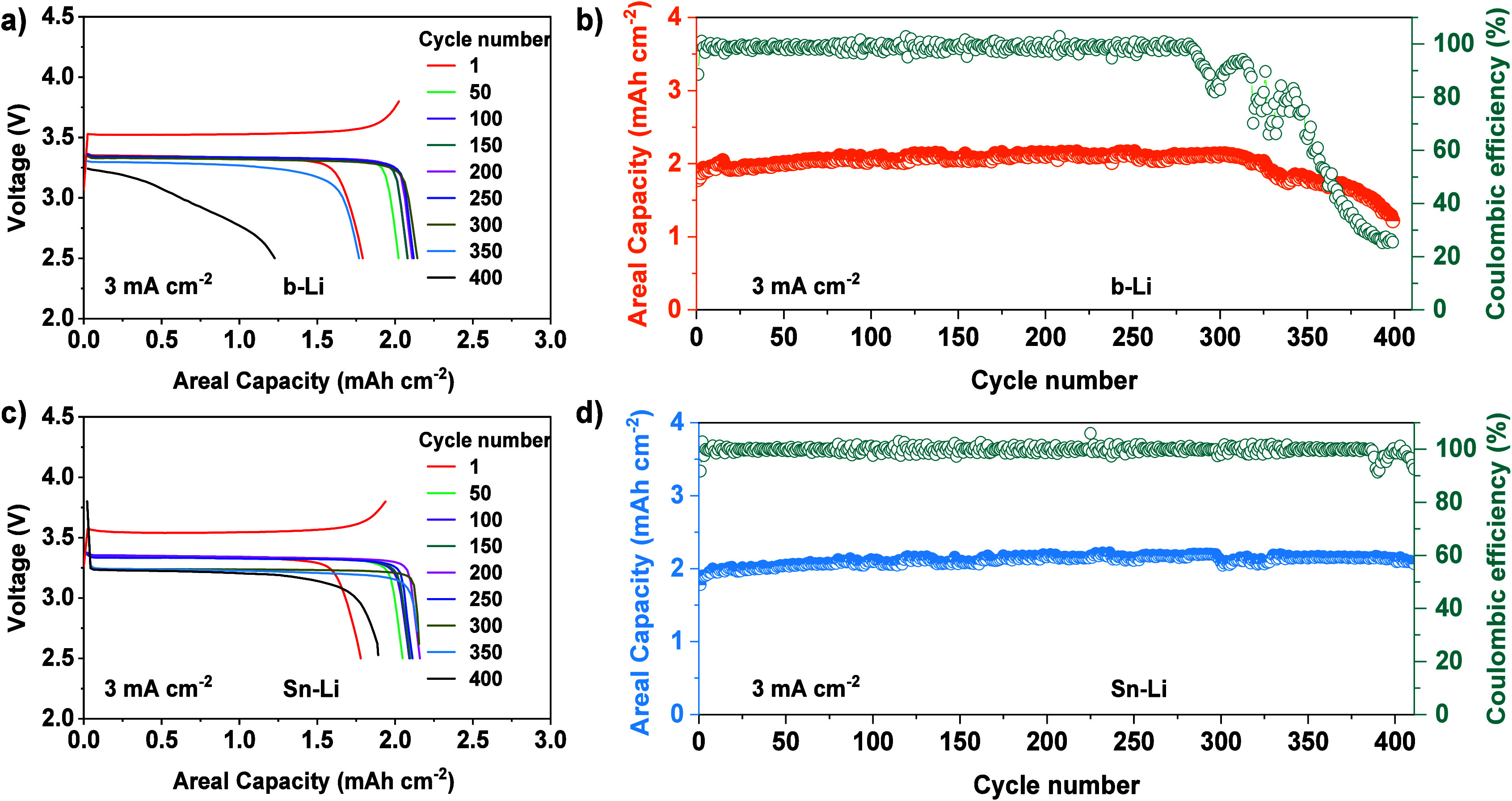
Full cell, LiFePO_4_ coupled with a lithium anode operated at 3 mA cm^–2^ current density using conventional ether electrolyte, 1 M LiTFSI DOL/DME + 2 wt % LiNO_3_ (1:1 v/v). Voltage profile and long-term cell performance for (a, b) b-Li anode and (c, d) Sn–Li anode, respectively.

**Figure 6:**
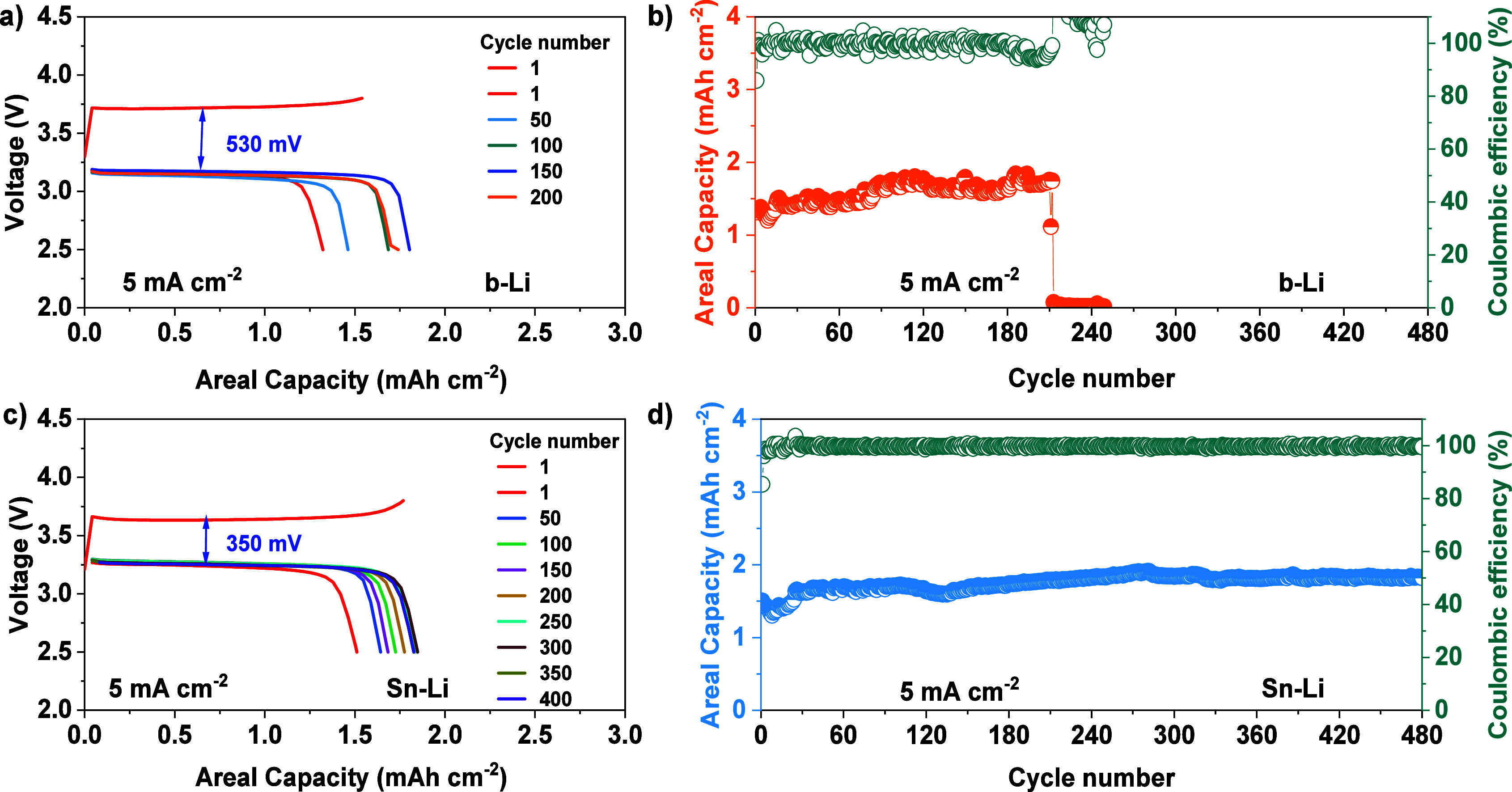
Full-cell performance of LiFePO_4_/x-Li (x = b or Sn) lithium anode operated at a high current density of 5 mA cm^–2^. Panels (a, b) and (c, d) represent the voltage profile and cyclic performance of b-Li and Sn–Li metal using ether electrolyte, respectively.

The overpotentials of the full cells, LiFePO_4_/b-Li and LiFePO_4_/Sn–Li at 5 mA cm^–2^, are determined as 530 and 350 mV, respectively, in the first cycle, indicating the beneficial effect of alloying on lithium reversibility, cyclic performance, and artificial LiCl-rich SEI formation in suppression of electrolyte decomposition, as shown in Figure 6. Additionally, cell performance was tested with a lower electrolyte amount (lean), and 20 μL of 1 M LiTFSI in DOL/DME + 2 wt % LiNO_3_ (1:1 v/v) was used to operate LiFePO_4_/Sn–Li cell at 0.5 mA cm^–2^. The cell demonstrates 100.87% retention capacity with an ACE of 100.47% after 110 cycles. The voltage profile and cyclability of the full cell are demonstrated in Figure S5a,b. Moreover, the versatility of lithium protection extended to a carbonate electrolyte (1 M LiPF_6_ in EC-DEC) in an LiNi_0.6_Mn_0.2_Co_0.2_O_2_/Li cell. The treated lithium, Sn–Li, under full-cell conditions, shows a capacity retention of 84.8% after 100 cycles, with an ACE of 99.6%. The notable benefit of Sn–Li alloying and LiCl SEI boosts the iCE to 87% compared to 78% in b-Li metal anode at 0.5 mA cm^–2^ current density, as shown in Figure S7. Fragile SEI, dendritic lithium growth, and dead lithium metal formation in b-Li result in 98.07% ACE and 81.3% retention capacity after 100 cycles under the same operational protocols.

As the current density increases, the reduction in capacity becomes noticeable. For this reason, Sn–Li coupled with a LiFePO_4_ cathode operated at 0.5 mA cm^–2^ showed superb stability, with 97.1% capacity retention after 360 cycles, as shown in Figure 7a,b. Furthermore, the rate capability of b-Li and Sn–Li, coupled with the LiFePO_4_ cathode, is shown in Figure 7c. Additionally, the rate capability test of the full-cell LiFePO_4_ cathode shows that at the lower current density, the discharge capacity and cyclability are indistinguishable for both b-Li and Sn–Li anodes. As the current density increases, the discharge capacity of b-Li decreases because of a possible interfacial reaction. A dramatic drop in capacity is observed as the high-current-density region shifts on bare lithium foil. The treated lithium anode on the other side showed outstanding rate capability, smooth reactivity, and unveiled stability, enabled by SnCl_4_ protection. The LiFePO_4_/Sn–Li battery shows consistent, symmetric, and asymmetrical cell performance with respect to the overpotential and lithium reversibility. Excellent cyclic performance at high current densities (3 and 5 mA cm^–2^), with superior retention capacitance and Coulombic efficiencies, demonstrates consistency and highlights the benefits of the dynamic protection mechanism in maintaining cycle stability. Moreover, studies on the LiCl-rich SEI and alloy formation are summarized in Table S1 for full-cell operation at different current densities and fabrication approaches, with respect to cycle life.

**Figure 7:**
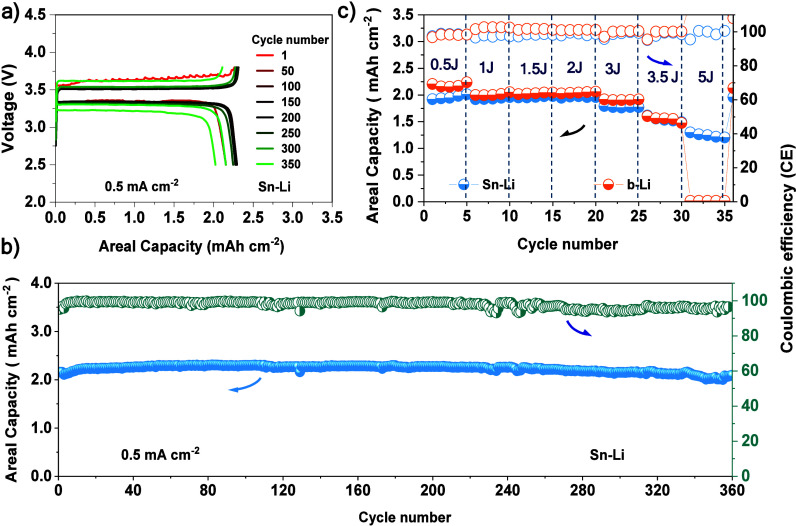
Full-cell and rate capability performance of LiFePO_4_ with a coupled lithium anode. (a, b) Voltage profile and cycle performance for Sn–Li anode, respectively, and (c) rate capability for LiFePO_4_/x-Li (x = b or Sn) using ether electrolyte.

Not only that, LTO (Li_4_Ti_5_O_12_) cathode was also operated at a current density of 0.5 mA cm^–2^. LTO/Sn–Li showed an iCE of 95.92% and was cycled for 130 cycles, with a retention capacity of 96.25% and an ACE of 99.8%, as indicated in Figure S8. This demonstrates ultra-stability and compatibility with lithium metal, reflecting the beneficial impact of artificial SEI formation from a sacrificial liquid salt.

An in situ electrochemical impedance spectroscopy (EIS) test was conducted under full-cell conditions (LiNi_0.6_Mn_0.2_Co_0.2_O_2_/x-Li, where x = b or Sn) to evaluate interfacial stability and enhance cell performance. Impedance was measured every 2 h as the cell was charged from 2.7 to 4.3 V vs Li/Li^+^. The Nyquist plots show a gradual change in impedance from the OCV state to the fully discharged state. Initially, at OCV, the impedance for both lithium electrode types is at its maximum, with b-Li showing higher values than Sn–Li, likely due to the electrolyte reacting with the fresh lithium–metal interface. The increase in impedance during the early charging stage aligns with operando heat-evolution measurements, which show higher heat flow at this stage. The impedance then decreases gradually, probably because of interface stabilization of the lithium anode in both cases. However, the impedance at the electrolyte/Sn–Li interface remains relatively low because of the formation of an artificial SEI, which helps reduce parasitic reactions at the electrode/electrolyte interface.

At the end of the discharge state, the impedance starts to grow, with the RC circuit forming a semicircle as the electrolyte reacts with the exposed lithium. The heat spikes from heat-evolution measurement demonstrated in Figure 4b,d correspond to the interface reaction as the exposed surface of lithium reacts with an electrolyte. The Nyquist plots and impedance alteration correspond to lithium-ion transport through the SEI film and to the charge-transfer impedance at the SEI/electrode interface. The formation of a resistive SEI at the Li/electrolyte interface leads to continuous capacity fading. The batteries with lower resistance exhibit better cyclability and reversibility, as confirmed in Figure S9. Interestingly, the electrolyte decomposes at the Li interface, leading to microstructural formation, as indicated by fluctuations in Coulombic efficiency, capacity drops, and decline. In the end, protective layers of an electronically conductive Sn–Li alloy, a lithium-ion-conductive LiCl interface, and a simple, efficient interface treatment substantially reinforce lithium reversibility and boost cycle life under an approach that delivers superior lithium metal battery design for high-energy-density batteries.

## Conclusions

In summary, a multifunctional lithium anode with artificial passivation, LiCl-rich SEI, and a Sn-lithium alloy enables dendrite-free lithium deposition and suppresses electrolyte decomposition at the Li/electrolyte interface. A facile and versatile solvent- and binder-free approach was employed, utilizing stannic chloride as a liquid salt. The protective layer facilitates uniform lithium deposition, ensuring dense, dendrite-free lithium growth. Synergistically, the contribution of the LiCl-rich SEI with Sn–Li alloy formation facilitates the homogeneous lithium deposition. Superior reversibility demonstrated in a symmetric Li|Li cell that operates for 600 h at a high current density and areal capacity of 5 mA cm^–2^/5 mAh cm^–2^, without an observed short-circuit. In a full-cell configuration, the protected lithium metal, when used as an anode, demonstrates outstanding capacity retention of 97.1% after 360 cycles at 0.5 mA cm^–2^, 100.3% after 410 cycles at 3 mA cm^–2^, and 93.8% after 480 cycles at 5 mA cm^–2^, with a LiFePO_4_ cathode. With a carbonate electrolyte, under LiNi_0.6_Mn_0.2_Co_0.2_O_2_/x-Li (x = b or Sn) at 0.5 mA cm^–2^, higher retention capacity was achieved as compared to the bare lithium metal anode. This approach demonstrates a solvent- and binder-free method for coating lithium surfaces, using a versatile brushing technique with a liquid salt to enable guided lithium growth, thereby enabling safe, high-performance lithium metal anodes.

## Supplementary Material







## Notes

Video support for NMC622/Li (MP4)
Video support for NMC622/Sn–Li (MP4)
Surface characterization, details of DFT studies, and electrochemical testing with LTO and NMC622 cathode (PDF)
