# Supplementary material for: Functional Interface Modifier with Visualizations of Dendrite Growth and Heat Evolution in Lithium Metal Batteries
Source: ACS Appl Mater Interfaces. 2026 Jun 23;18(26):36686–95. doi: 10.1021/acsami.5c25165 (PMC13352515; doi:10.1021/acsami.5c25165)
Supplement: Supplementary file 3 [file am5c25165_si_003.pdf]

Supporting Information

Functional interface modifier with visualizations of dendrite growth and heat evolution in lithium metal batteries

Bereket Woldegbreal Taklu,[a,b] Tsung-I Yeh,[a] Ashok Vallal Saravanan,[a,c] Elango Balaji Tamilarasan,[a] Siyanand Kumar Chaudhary,[a,c] Zabish Bilew Muche,[a] Boligarla Vinay,[a] Teshome Gonfa Hordofa,[a,c] Kidane Goitom Gerezgiher,[a,c] Chung Ray Weng,[a] Tzu-Ting Hung,[a] Sheng-Chiang Yang,[a] Wei-Nien Su,*[b,d], and Bing Joe Hwang,*[a,b,e]

[a]Nano-electrochemistry Laboratory, Department of Chemical Engineering, National Taiwan University of Science and Technology, Taipei 106, Taiwan

[b]Sustainable Electrochemical Energy Development Center, National Taiwan University of Science and Technology, Taipei City 106, Taiwan

[c]Battery Research Center of Green Energy, Ming Chi University of Technology, New Taipei City, 24301, Taiwan

[d]Nano-electrochemistry laboratory, Graduate Institute of Applied Science and Technology, National Taiwan University of Science and Technology, Taipei 106, Taiwan

[e]National Synchrotron Radiation Research Center (NSRRC), Hsin-Chu, 30076, Taiwan

*Corresponding Authors:

wsu@mail.ntust.edu.tw
bjh@mail.ntust.edu.tw

Details of the DFT computation study

To study lithium-ion diffusion in the Li and Li_5Sn_2 systems, we performed first-principles calculations using the Vienna Ab-initio Simulation Package (VASP).[1-2] The calculations employed projector augmented wave (PAW) pseudopotentials,[3] and the electronic exchange-correlation effects were described using the generalized gradient approximation (GGA),[4] within the Perdew-Burke-Ernzerhof (PBE) functional framework.[5] For surface property analysis, we examined various crystallographic orientations, including (100), (010), (001), (101), (110), (011), and (111), for each material. After determining the most stable surface configurations, we constructed slab models with 3×3×1 supercells for the Li(001) and Li_5Sn_2(111) surfaces. These were used to assess lithium diffusion by tracking atomic migration between energetically favorable adsorption sites. To evaluate the diffusion energy barriers, we employed the climbing-image nudged elastic band (c-NEB) method.[6-7] A Monkhorst-Pack k-point mesh of 5×5×2 was utilized for Brillouin zone sampling. Atomic structures were relaxed until the force on each atom was below 0.02 eV/Å, ensuring precise determination of activation energies.[8-9] Our analysis focused on direct lithium hopping within the same atomic plane, modeling ion migration between adjacent lithium sites to accurately quantify the diffusion barriers.

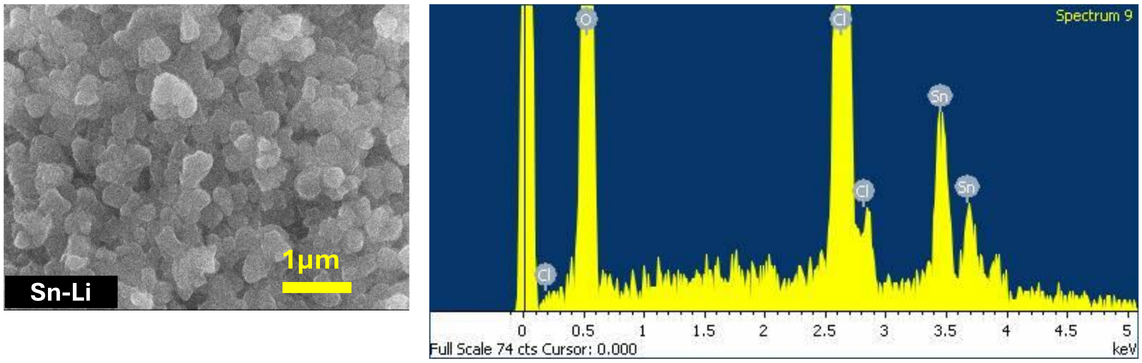

Figure S1. Surface microstructural examination of Sn-Li metal and its EDS analysis for Sn and Cl.

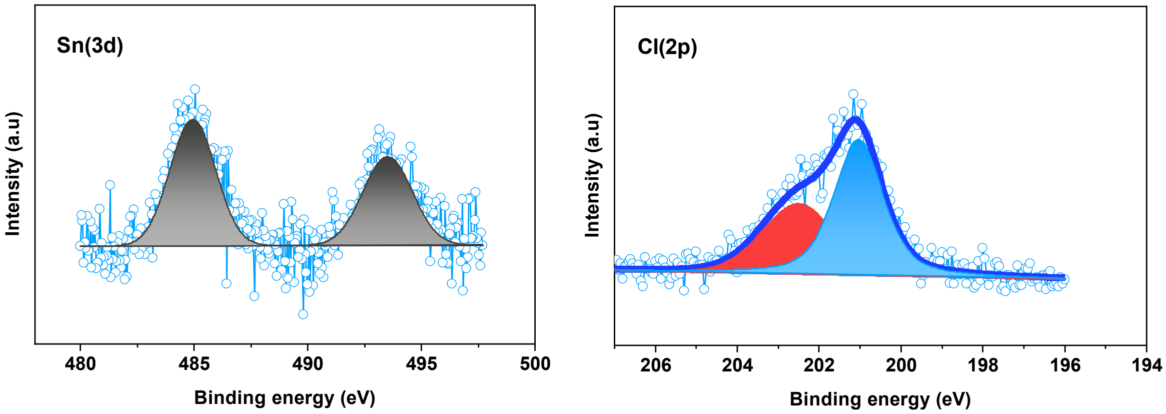

Figure S2. XPS spectra for $SnCl_4$-treated lithium surface, Sn-Li at Sn(3d) and Cl(2p) coreshells.

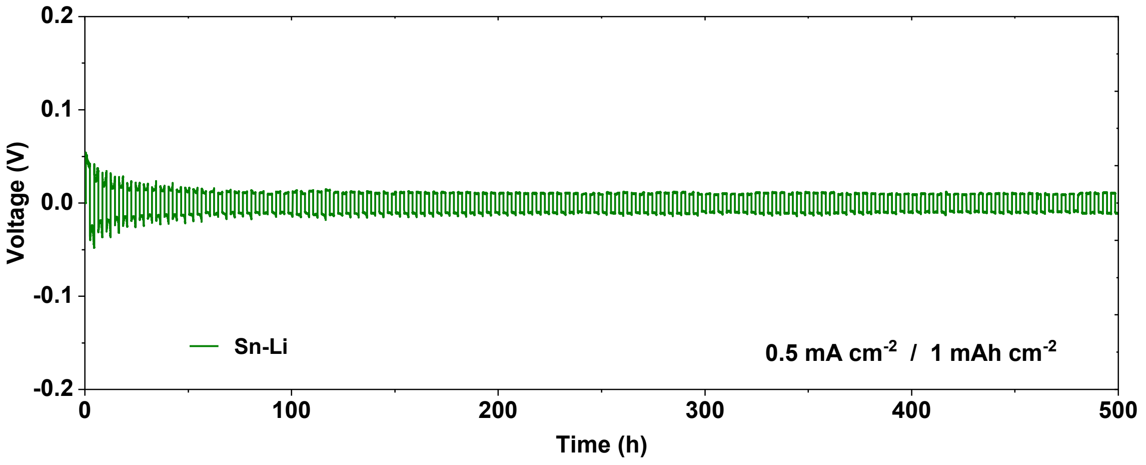

Figure S3. Symmetrical cell tested for Sn-Li ‖ Sn-Li operated at current density of 0.5 mA cm^{-2} / 1 mAh cm^{-2} using 1 M LiTFSI DOL-DME + 2 wt% $LiNO_3$ electrolyte.

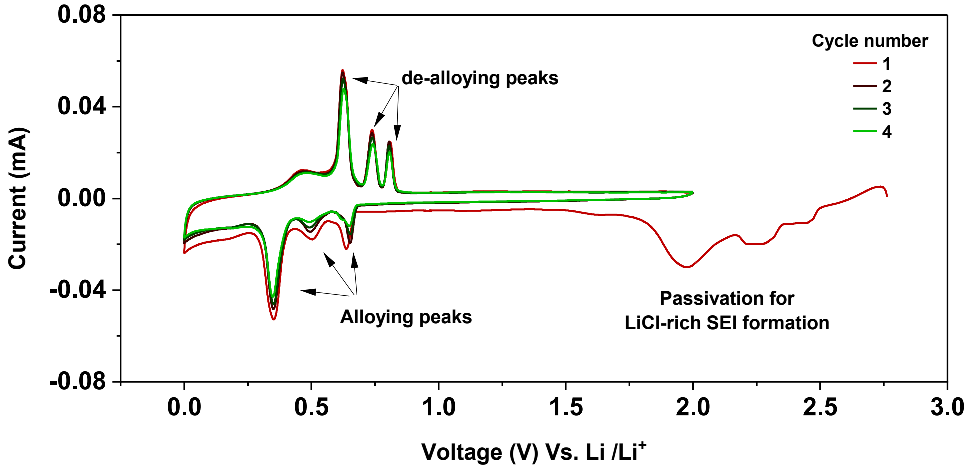

Figure S4. Cyclic voltammetric measurement for asymmetrical cell, Li/ Cu, performed at 0.1 mV s^{-1} scan rate using 1 M $LiPF_6$ EC-DEC + 3 v/v % $SnCl_4$ electrolyte.

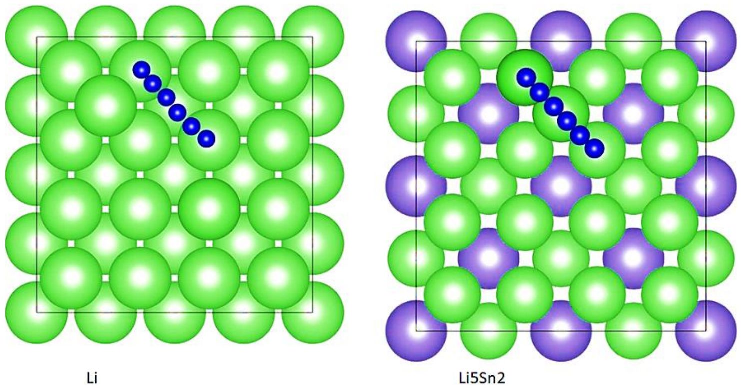

Figure S5: Diffusion paths and barriers of Li on various surfaces: (a) Li; (b) Li_5Sn_2. (Green spheres depict Li atoms, light gray spheres designate Sn atoms, and continuous blue lines denote Li diffusion paths)

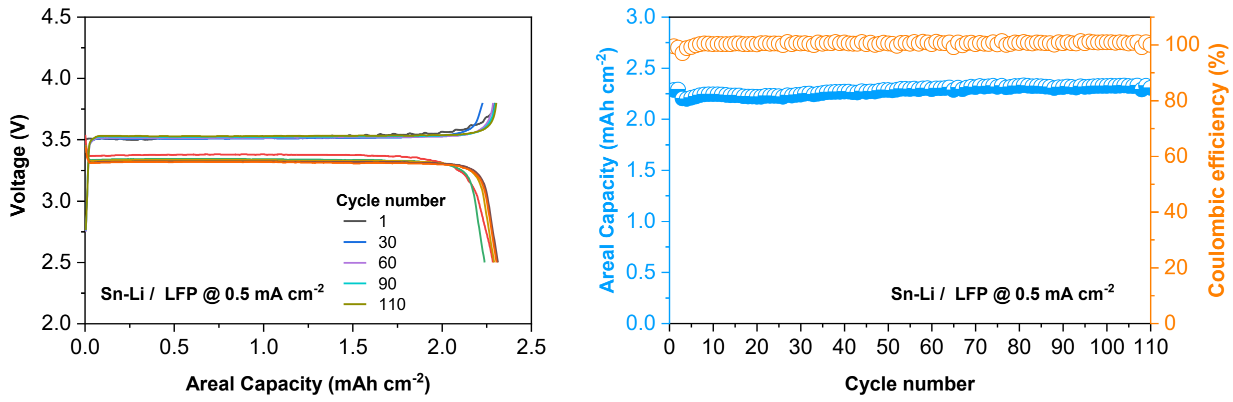

Figure S6. Galvanostatic voltage profile (a) and cyclic performance (b) of LFP/Sn-Li operated at 0.5 mA cm^{-2} for under 1 M LiTFSI DOL/DME + 2 wt. $LiNO_3$ (1:1 v/v) electrolyte amount using 20 μL.

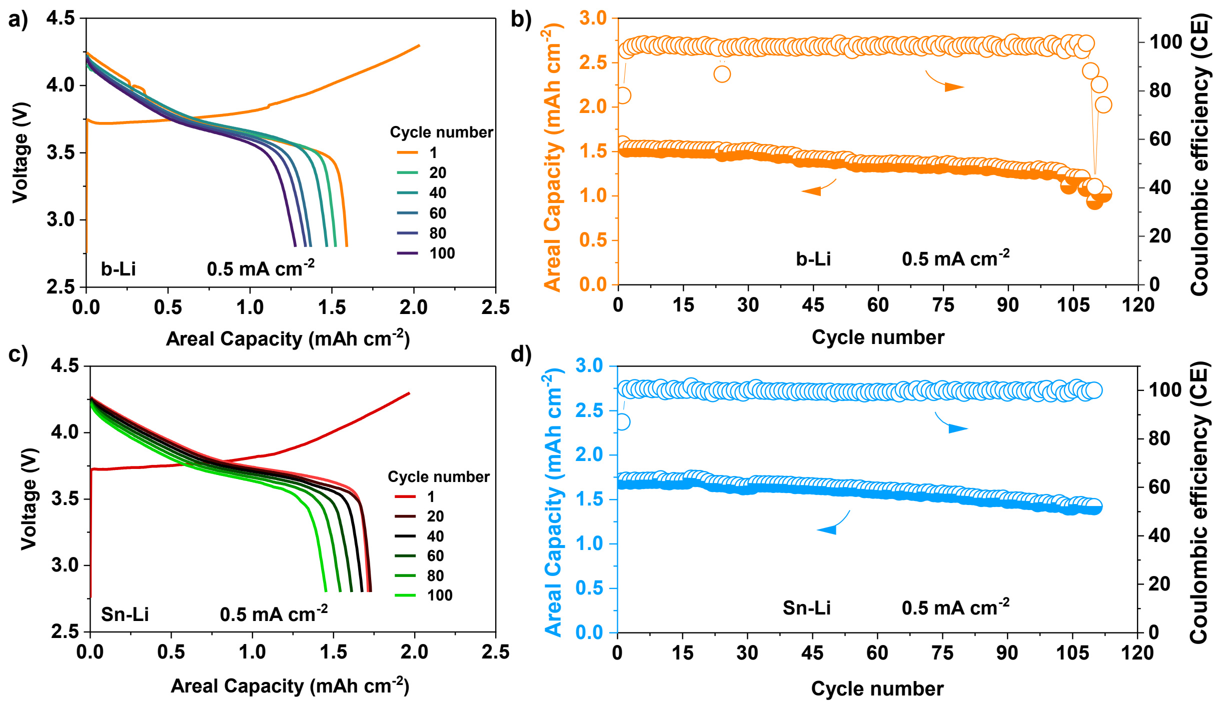

Figure S7. Full-cell performance for $LiNi_{0.6}Mn_{0.2}Co_{0.2}O_2$ / x-Li (x = b or Sn) operated at 0.5 mA cm^{-2} using 1 M $LiPF_6$ EC-DEC electrolyte. a) and b) correspond to the voltage profile and long-term performance of the b-Li anode. c) and d) correspond to the voltage profile and long-term performance of the Sn-Li anode.

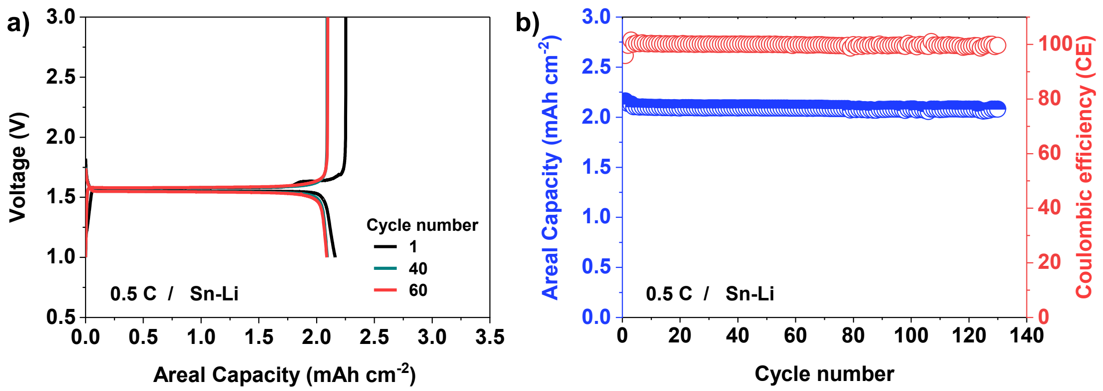

Figure S8. Full cell, LTO / Sn-Li operated at 0.5 mA cm^{-2} current density under 1 M LiTFSI DOL-DME + 2 wt% $LiNO_3$ electrolyte. a) voltage profile and b) cyclic performance of LTO / Sn-Li cell.

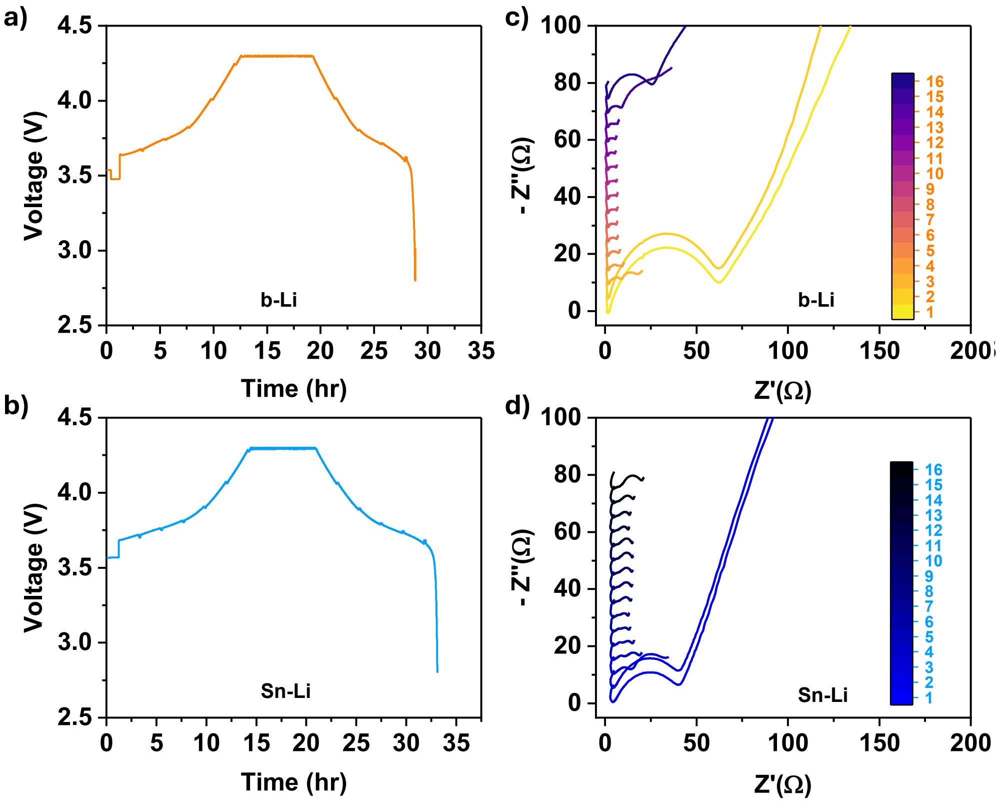

Figure S9. An in-situ EIS measurement for a full cell, $LiNi_{0.6}Mn_{0.2}Co_{0.2}O_2$ / x-Li (x = b or Sn) for interface resistance evolution analysis. a) Voltage-time curve for $LiNi_{0.6}Mn_{0.2}Co_{0.2}O_2$ / x-Li (x = b or Sn), and c) and d) correspond to their Nyquist plot, respectively.

Table S1. The comparative illustration of chlorine-rich SEI with/out metallic seeding under solvent-free and solvent-based approaches is presented in the current work.

Interface stabilizers	*Approaches used (Solvent or solvent-free)*	*Electrolyte*	*Current density*	*Full cell configurations*	*Cycle life/retention capacity*	*Ref.*
**$AlCl_3$, $CuCl_2$, $ZnCl_2$**	NMP solvent	1 M $LiPF_6$ in EC/DEC	5 C	$LiFePO_4$ / $CuCl_2$-Li	2000/ 85.6%	[10]
**$AlCl_3$**	THF solvent	1 M LiTFSI DOL: DME + 2 wt% $LiNO_3$	1 C	$LiFePO_4$ / $AlCl_3$-Li	300 / 74.8%	[11]
**$C_8H_{17}Cl$**	THF solvent	1 M LiTFSI DOL: DME + 2 wt% $LiNO_3$	2 C	$Li_4Ti_5O_{12}$ / LiCl-Li	1000 / 65.8	[12]
**$SnCl_4$**	Solvent-free	1 M LiTFSI DOL: DME + 2 wt% $LiNO_3$	3 mA cm $^{-2}$<br>5 mA cm $^{-2}$	$LiFePO_4$ / $SnCl_4$-Li	410 / 100.3 % (3 mA cm^{-2})<br>480/93.8 % (5 mA cm^{-2})	**This work**

References

1. Kresse, G.; Hafner, J., Ab initio molecular dynamics for liquid metals. *Physical review B* **1993,** *47* (1), 558.
2. Kresse, G.; Furthmüller, J., Efficient iterative schemes for ab initio total-energy calculations using a plane-wave basis set. *Physical review B* **1996,** *54* (16), 11169.
3. Blöchl, P. E., Projector augmented-wave method. *Phys. Rev. B* **1994,** *50* (24), 17953.
4. Perdew, J. P.; Burke, K.; Ernzerhof, M., Generalized gradient approximation made simple. *PHYS REV LETT* **1996,** *77* (18), 3865.
5. Perdew, J. P.; Ruzsinszky, A.; Csonka, G. I.; Vydrov, O. A.; Scuseria, G. E.; Constantin, L. A.; Zhou, X.; Burke, K., Restoring the density-gradient expansion for exchange in solids and surfaces. *PHYS REV LETT* **2008,** *100* (13), 136406.
6. Henkelman, G.; Jónsson, H., Improved tangent estimate in the nudged elastic band method for finding minimum energy paths and saddle points. *The Journal of chemical physics* **2000,** *113* (22), 9978-9985.
7. Henkelman, G.; Uberuaga, B. P.; Jónsson, H., A climbing image nudged elastic band method for finding saddle points and minimum energy paths. *The Journal of chemical physics* **2000,** *113* (22), 9901-9904.
8. Startt, J.; Deo, C.; Dingreville, R., Vacancy surface migration mechanisms in dilute nickel-chromium alloys. *Scripta Materialia* **2021,** *202*, 113998.
9. Restrepo, O. A.; Becquart, C. S.; El-Mellouhi, F.; Bouhali, O.; Mousseau, N., Diffusion mechanisms of C in 100, 110 and 111 Fe surfaces studied using kinetic activation-relaxation technique. *Acta Materialia* **2017,** *136*, 303-314.

10. Qian, S.; Xing, C.; Zheng, M.; Su, Z.; Chen, H.; Wu, Z.; Lai, C.; Zhang, S., CuCl2-Modified Lithium Metal Anode via Dynamic Protection Mechanisms for Dendrite-Free Long-Life Charging/Discharge Processes. *Advanced Energy Materials* **2022,** *12* (15), 2103480.

11. Hou, G.; Ci, C.; Guo, H.; Zhang, X.; Sun, Q.; Cheng, J.; Salpekar, D.; Ai, Q.; Chen, L.; Puthirath, A. B.; Kato, K.; Pardo, S. C.; Vajtai, R.; Babu, G.; Ci, L.; Ajayan, P. M., Facile construction of a hybrid artificial protective layer for stable lithium metal anode. *Chemical Engineering Journal* **2020,** *391*, 123542.

12. Tan, L.; Chen, Q.; Chen, P.; Huang, X.; Li, L.; Zou, K.; Liu, D., Lithium chloride protective layer for stable lithium metal anode via a facile surface chemistry. *Journal of Electroanalytical Chemistry* **2023,** *928*, 117063.
